# Hyperammonaemic Encephalopathy Caused by Adult-Onset Ornithine Transcarbamylase Deficiency

**DOI:** 10.3390/brainsci12020231

**Published:** 2022-02-08

**Authors:** Bjarke Hammer Niclasen, Maria Therese Schelde-Olesen, Mads Astvad, Anders Løkke, Thomas Krøigård, Helle H. Nielsen

**Affiliations:** 1 Department of Neurology, Odense University Hospital, J.B. Winsloewsvej 4, 5000 Odense, Denmark; Bjarke.Hammer.Niclasen2@rsyd.dk (B.H.N.); thomas.kroigard@rsyd.dk (T.K.); 2 Department of Clinical Genetics, Odense University Hospital, J.B. Winsloewsvej 4, 5000 Odense, Denmark; Maria.T.Schelde-Olesen@rsyd.dk; 3 Department of Intensive Care, Odense University Hospital, J.B. Winsloewsvej 4, 5000 Odense, Denmark; mads.astvad@rsyd.dk; 4 Department of Medicine, Little Belt Hospital, 7100 Vejle, Denmark; anders.lokke@rsyd.dk; 5 Department of Regional Health Research, University of Southern Denmark, 5230 Odense, Denmark; 6 Department of Neurobiology Research, Institute of Molecular Medicine, University of Southern Denmark, J.B. Winsloewsvej 21, 5000 Odense, Denmark; 7 BRIDGE-Brain Research—Inter-Disciplinary Guided Excellence, Department of Clinical Research, J.B. Winsloewsvej 19, 5000 Odense, Denmark

**Keywords:** subacute encephalopathy, hyperammonaemia, ornithine transcarbamylase deficiency, adult onset, brain oedema

## Abstract

Hyperammonaemic encephalopathy in adults is a rare condition in the absence of liver disease and is associated with a high mortality and risk of permanent neurological deficits. Seldomly, the condition is caused by an inborn error of metabolism in the urea cycle, triggered by an exogenic factor such as gastrointestinal haemorrhage, gastric bypass surgery, starvation, seizures, vigorous exercise, burn injuries, or drugs hampering the elimination of ammonia. Here, we present a fatal case of an unrecognized genetic ornithine transcarbamylase deficiency (OTCD) presenting with a subacute progressive encephalopathy. We review the current literature and discuss the differential diagnosis and treatment options. As swift diagnosis and initiation of treatment is vital, awareness of hyperammonaemic encephalopathy and its possible causes can help improve the prognosis of this condition.

## 1. Introduction

Hyperammonaemic encephalopathy is a broadly defined, neuro-psychiatric condition in which brain function is affected, typically with a reduced level of consciousness [1]. Most adult cases of hyperammonaemia are caused by liver cirrhosis or acute liver failure [2], but it can occur in patients with normal liver function [3]. In these circumstances, the condition is almost always caused by one or more triggering factors [1,4] although unexplained cases also exist [5].

Triggering factors can be divided into two major categories:

(I) An overproduction of ammonia due to: (i) excessive protein load originating from conditions such as gastrointestinal haemorrhage, gastric bypass surgery, multiple myeloma, allogenic stem cell transplantation, or parenteral nutrition; (ii) an increased metabolism caused by starvation, seizures, vigorous exercise, burn injuries, or corticosteroid treatment; or (iii) urinary overproduction caused by urease-producing infections [1,2,3].
(II) A reduced elimination of ammonia due to (i) liver failure caused by hepatitis or alcohol abuse; (ii) drugs such as antiepileptic drugs [6,7,8,9,10,11], pain medication [11,12], acetazolamide [13], chemotherapy [14], and methamphetamine [15]; or (iii) inborn errors of metabolism (urea cycle disorders (UCDs), organic acidaemias or fatty acid oxidation disorders) [16].

Symptoms of hyperammonaemia range from mild (nausea, vomiting, headache) to severe encephalopathy including behavioural changes, seizures, and decreased consciousness. Untreated, the condition can lead to brain oedema, ultimately resulting in a fatal outcome [3,5].

**Figure 1:**
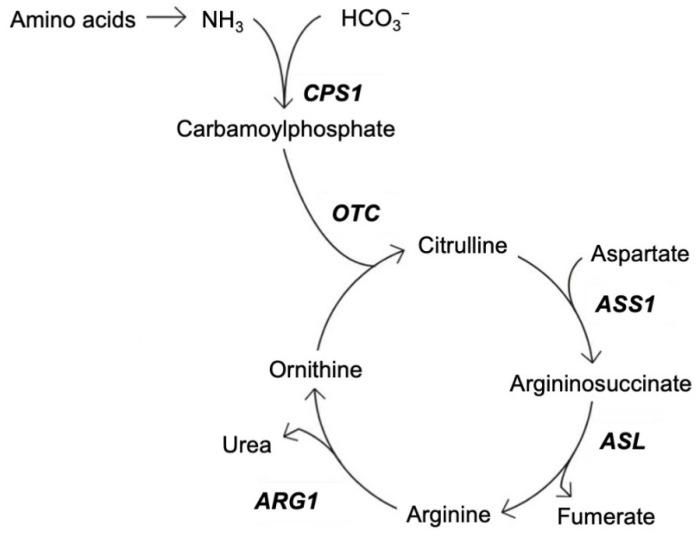
Schematic overview of the urea cycle. CPS1—carbamoyl phosphatase synthetase; OTC—ornithine transcarbamylase; ASS1—argininosuccinate synthase 1; ASL—argininosuccinate lyase; ARG1—arginase 1. Adapted from [4,18].

Ammonia is continuously produced in the human body by several organs, including the muscle tissue and kidneys, but most is derived from the gastrointestinal system [4,17]. Through the portal circulation, ammonia metabolized from the small intestine is transported to the liver and degraded (Figure 1) [4].

While some of the ammonia is reutilized through biosynthesis, the remainder is primarily excreted in the urine as urea. Ammonia in the extrahepatic tissue is incorporated into the non-toxic amino acid, glutamine [4,17]. The process of nitrogen clearance through the urea cycle (Figure 1) is mainly limited to two enzymes: carbamoyl phosphate synthetase (CPS1) and ornithine transcarbamylase (OTC) which is found in the human liver, although extra-hepatic OTC also exists to a lesser degree [19]. Ammonia can freely cross the blood–brain barrier (BBB), and at high concentrations, has a toxic effect on brain tissue [2,17]. The cerebrospinal fluid (CSF) contains approximately one-third to one-half of the ammonium content found in arterial blood. Several diseases and their inherited hyperammonaemias can increase the ammonium content of the CSF, with the severity of encephalopathy correlating with the ammonium content [20].

UCDs are a subgroup of diseases caused by inborn errors of the metabolism [21]. UCDs have a significant morbidity due to acute and chronic neurotoxicity that is often associated with massive hyperammonaemia, potentially resulting in brain oedema [17,21]. OTCD is the most common UCD known to cause hyperammonaemia [21,22]. The prevalence of OTCD is estimated to be 1 in 62,000 to 77,000 live births. However, this prevalence is biased towards the earliest and most severe presentations, as OTCD can manifest at any age [23]. Given the relative rarity of the condition, adult patients with OTCD and other UCDs are, thus, at risk of being underdiagnosed [24].

Here, we present a fatal case of unrecognized OTCD and discuss relevant investigations, treatment options, and differential diagnosis.

## 2. Case Report

This case was a 48-year-old man with no family history of liver disease or metabolic disorders and no history of alcohol abuse, liver failure, surgery, or dietary changes.

**Figure 2:**
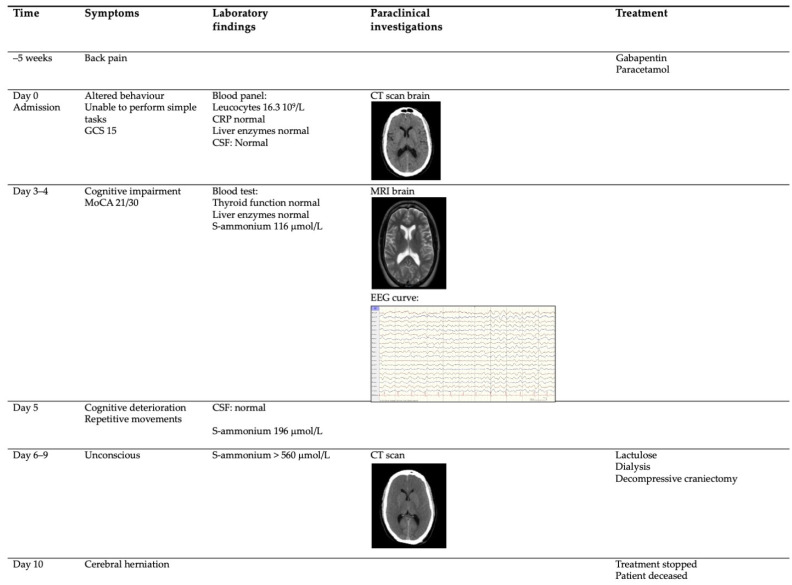
Schematic overview of the case. The patient had developed back pain 5 weeks prior to this hospital admission to investigate altered behaviour. Treatment was stopped due to cerebral herniation 10 days after admission. GCS—Glasgow Coma Score; MoCA—Montreal Cognitive Assesment score; CSF—cerebrospinal fluid; S—serum; CRP—C reactive peptide.

Five weeks prior to hospital admission, the patient suffered from lower back pain and was diagnosed with a disc herniation, and gabapentin and paracetamol were prescribed as pain medication (Figure 2, -5 weeks). There was no prior rigorous exercise.

The patient was referred to the emergency room by his general practitioner, because the family reported they had noticed behavioural changes. The patient was unable to perform simple, everyday tasks such as tying shoelaces and buckling a seat belt, and was only communicating in short, simple sentences. There were no complaints of nausea or vomiting. The neurological examination was normal with a Glasgow coma score of 15, although the patient had to be repeatedly instructed to comply with the neurological examination.

A standard panel of blood tests revealed leucocytosis (16.3 10^9^/L), normal CRP, and normal liver enzymes. The computed tomography (CT) of the brain was normal, and the initial CSF was unremarkable with no pleocytosis (Figure 2, Day 0).

Over the following days, 1–3 (Figure 2, Day 3–4), the patient’s cognitive abilities deteriorated. The Montreal Cognitive Assessment score was 21/30 points. Blood tests excluded thyroid abnormalities, HIV, and syphilis. Serum ammonia was measured as 116 µmol/L, while aspartate transaminase (AST), alanine aminotransferase (ALT), gamma-glutamyl transferase (GGT), bilirubin, lactate dehydrogenase, and international normalized ratio of prothrombine time (INR) were all within normal ranges. A repeated lumbar puncture showed normal leucocyte and protein levels and no oligoclonal bands. There were no antibodies suggestive of neuroinfection, paraneoplastic conditions, or autoimmune encephalitis. Magnetic resonance imaging (MRI), including gadolinium of the brain and spinal cord, was normal (Figure 2, Day 3–4). The electroencephalogram (EEG) was dominated by diffuse 4–5 Hz of activity with an amplitude of approximately 40 µV. The frequency and amplitude were symmetrical. Intermittent synchronous and asynchronous 2–3 Hz of activity was observed over the temporo–frontal regions with the occasional spread to the posterior regions. Furthermore, short trains of diffuse rhythmic delta activity (2.5–3 Hz) with a frontal predominance and a duration of 3–4 s were observed (Figure 2, Day 3–4). EEG findings were consistent with moderate, diffuse cerebral dysfunction. A CT scan of the abdomen was performed to rule out liver abnormalities including portosystemic shunt conditions.

On day 5, the patient’s cognitive status had deteriorated further. He was no longer able to answer questions relevantly. He developed repetitive muscular movement (Figure 2, Day 5).

On days 6–9 the patient was transferred to the intensive care unit and rendered unconscious. Serum ammonia had now risen to >560 µmol/L, and the brain CT showed signs of increasing intracranial pressure (ICP) and cerebral oedema (Figure 2, Day 6–9).

A decompressive bifrontal craniectomy was performed without the sufficient lowering of ICP, measured by an intracranial pressure screw. Although both dialysis and lactulose were initiated, cerebral herniation occurred, and the patient was pronounced dead 10 days after admission.

The autopsy revealed no underlying structural organ abnormalities. However, genetic analysis showed the patient to be hemizygous for c.672C > A; p.Pro225Thr in the OTC gene. The suspicion of metabolic UCD was thereby confirmed. The family members were all offered a genetic analysis.

## 3. Genetic Analysis

The OTC gene is inherited in an X-linked recessive manner. Most frequently, patients with OTCD are hemizygous males, but approximately 20% of female carriers also show symptoms due to skewed X-chromosome inactivation. If the female parent is a carrier, male offspring have a 50% risk of inheriting the disease.

A mutation analysis of the OTC gene showed that the patient was hemizygous for c.672C > A; p.Pro225Thr in the OTC gene, a known pathogenic variant [25,26]. This variant has previously been described in an otherwise healthy 62-year-old man with late-onset presentation, wherein genetic analysis showed a Pro-225-Thr (P225T) change, the same as in our patient. This confirms that the Pro225Thr variant, in more than one case, has been associated with late-onset OTC deficiency [26].

**Figure 3:**
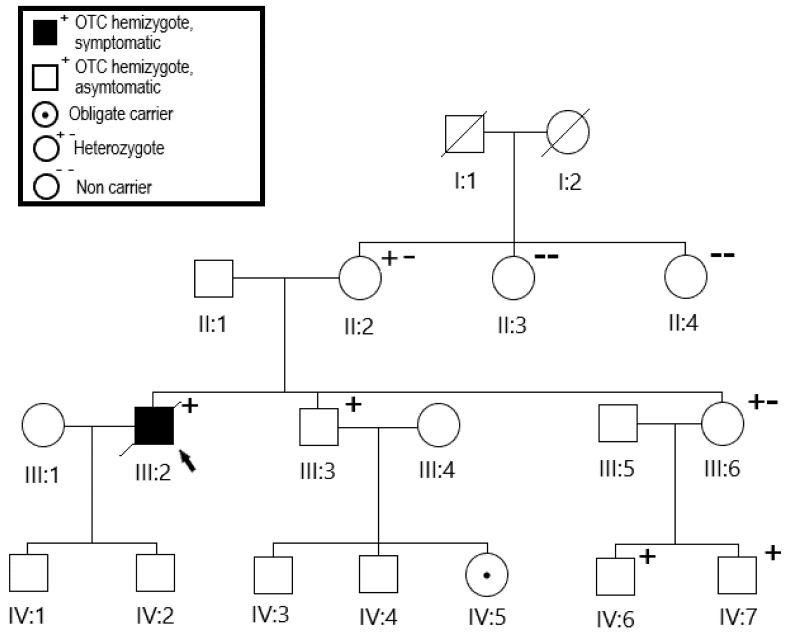
Family tree. Mutation analysis of the family showed the brother’s daughter (IV:5) to be an obligate carrier. The sister’s children (IV:6,7) were all found to be carriers. Oblique line through the box indicates deceased individual. Squares indicate males, and circles indicate females.

The genetically confirmed diagnosis made family testing possible, which is crucial to avoid other fatal cases (Figure 3).

By family testing, individual II:2 was found to be heterozygote. As the variant is inherited in an X-linked manner III:3 and III:6 each had a 50% risk of having inherited the variant. The variant was detected in both patients. Individual IV:5 was then an obligate carrier. Individuals IV:6 and IV:7 were also tested and were both hemizygote. Several other family members underwent mutation analysis. Seven family members have tested positive so far, and they have so far been asymptomatic. Prenatal diagnosis in this family is now possible because of the known disease-causing mutation.

## 4. Discussion

We present a case of hyperammonaemic encephalopathy without liver failure that was caused by an unrecognized OTC deficiency. The initial presentation with subacute, progressive encephalopathy over days made the diagnosis difficult, as the causes of this are numerous [27]. Early on, an EEG confirmed encephalopathy, and investigations concentrated on the underlying cause.

With no history of excessive alcohol intake, chemotherapy, lithium, or antiepileptic drugs, toxic causes could be ruled out. Infectious causes of encephalopathy were ruled out by blood tests, neuroimaging, and CSF investigations. Neuroinflammatory causes of encephalopathy, such as autoimmune encephalitis, were briefly suspected but were ruled out by the normal MRI without gadolinium enhancement and the CSF devoid of antibodies, pleocytosis, and oligoclonal bands. A positron-emission tomography (PET) scan of the brain was planned, but was cancelled due to deterioration in the patient’s clinical state. An underlying vascular cause of encephalopathy was inconsistent with the subacute presentation and was excluded by the normal MRI, just as underlying CNS malignancies were ruled out. Neuropsychiatric causes of encephalopathy include dementia, depression, and psychotic episodes [12]. In this case, the subacute progression, the patient’s age, and the absence of a psychiatric history made a neuropsychiatric condition unlikely.

The high levels of ammonia led to the suspicion of an underlying metabolic disorder. Hyperammonaemia can be caused by either overproduction of ammonia or reduced elimination. Despite a detailed history, there were no indications of underlying conditions leading to overproduction such as changes in diet or exercise pattern, gastrointestinal haemorrhage, infection, organ transplantation, or parenteral nutrition.

The most common cause of reduced elimination of ammonia is liver failure due to liver cirrhosis, acute liver failure, or portosystemic shunts [2]. This patient had no history of excessive alcohol intake, and liver enzymes were normal with no sign of hepatitis. A CT revealed no liver abnormalities. Ammonia elimination can also be hampered by certain drugs. The most common is valproic acid [28], but hyperammonaemia can also be caused by carbamazepine [29], topiramate [8], lamotrigine [9], primidone [10], gabapentin [11], salicylates [12], acetazolamide [13], chemotherapy [14], and methamphetamine [15]. Prior medication for this patient only included the temporary use of paracetamol and gabapentin, not deemed toxic to the liver.

Despite no family history, genetic investigations revealed a family tree of a hitherto asymptomatic mutation in the OTC gene. Whereas many genetic UCDs are diagnosed in the neonatal period or early childhood, a partial defect in the OTC gene is associated with adult-onset disease [30]. Although men often have an earlier presentation and a more severe course of the disease, healthy adult males can present with a hyperammonaemic crisis as seen in the current case [30,31]. Triggers often include well-known factors of hyperammonaemia, none of which were present in this case. Prior to admission, the patient was prescribed paracetamol and gabapentin for back pain. Gabapentin has been known to cause hyperammonaemia and toxic encephalopathy in the absence of liver failure [11]. The paracetamol dose was low, but it is possible that it contributed through a subclinical liver inflammation, and thereby reduced liver function as seen in other UCDs [18]. However, it is striking that no liver abnormalities were detected in the blood tests, the CT scan, or at autopsy.

The rapid progression and fatal outcome of this case underlines the necessity for a swift and comprehensive differential diagnosis in cases of progressive encephalopathy of unknown cause, especially in cases where treatment of the underlying cause is available. Serum ammonia should always be measured in the initial workup [24,31] despite the risk of false-positive results [32]. Analyses of blood or urine amino acids such as arginine, citrulline or ornithine analysis could have potentially contributed to a faster diagnosis. Retrospective studies of hyperammonaemic encephalopathy have shown that most cases are caused by acquired exogenic factors such as malnutrition or infection, while urea cycle disorders are rare causes with incidences ranging from 4–13% [3,5]. Adult-onset hyperammonaemic encephalopathy has a mortality rate of 30–39% [3,5,33] and a high risk of neurological deficits among the survivors [5]. A swift initiation of therapy is essential for the outcome, and could potentially have changed the outcome of this case. Danish recommendations include a decreased protein intake and the administration of intravenous glucose to prevent catabolism and protein breakdown [33]. Ammonia levels can effectively be lowered by dialysis [3,5,33] or the administration of ammonium-lowering drugs such as sodium benzoate, glycerol phenylbutyrate, or sodium phenylbutyrate, but these should be prescribed under guidance of a specialized centre for metabolic disorders [18]. Furthermore, L-arginine hydrochloride and L-carnitine can be administered depending on the type of urea cycle defect [3,5,24,33].

## 5. Conclusions

We present a patient with subacute hyperammonaemic encephalopathy associated with a previously undiagnosed urea cycle defect. As the fatal outcome shows, a swift and accurate diagnosis and rapid initiation of treatment is vital. While treatment guidelines are scarce [34], prompt initiation of haemodialysis and other ammonium-lowering treatments is essential and should be instigated in cases of hyperammonaemic encephalopathy regardless of the underlying condition. Since this is a rare condition, the diagnosis and treatment should preferably be carried out in consultation with a specialized centre for metabolic disorders, as soon as the suspicion arises. Awareness of hyperammonaemic encephalopathy and its possible causes can help to improve the prognosis of this condition.

## Data Availability

The data presented in this study are available on request from the corresponding author. The data are not publicly available due to Danish data protection regulations.
